# The association between gut microbiome and PCOS: evidence from meta-analysis and two-sample mendelian randomization

**DOI:** 10.3389/fmicb.2023.1203902

**Published:** 2023-07-24

**Authors:** Qiusi Min, Hongling Geng, Qian Gao, Min Xu

**Affiliations:** ^1^Guangzhou University of Chinese Medicine, Guangzhou, Guangdong, China; ^2^Department of Gynecology, Guangdong Provincial Hospital of Chinese Medicine, Guangzhou, Guangdong, China

**Keywords:** Mendelian randomization, PCOS (polycystic ovarian syndrome), gut microbiome, MiBioGen, SNP, genetics

## Abstract

**Background:**

Increasing evidence from observational studies and clinical experimentation has indicated a link between the gut microbiotas (GMs) and polycystic ovary syndrome (PCOS), however, the causality and direction of causality between gut microbiome and PCOS remains to be established.

**Methods:**

We conducted a comprehensive search of four databases–PubMed, Cochrane Library, Web of Science, and Embase up until June 1, 2023, and subjected the results to a meta-analysis. In this study, a bidirectional two-sample Mendelian randomization (MR) analysis was employed to investigate the impact of gut microbiota on polycystic ovary syndrome (PCOS). The genome-wide association study (GWAS) data for PCOS comprised 113,238 samples, while the GWAS data for gut microbiota were derived from the MiBioGen consortium, encompassing a total sample size of 18,340 individuals. As the largest dataset of its kind, this study represents the most comprehensive genome-wide meta-analysis concerning gut microbiota composition to date. Single nucleotide polymorphisms (SNPs) were selected as instrumental variables at various taxonomic levels, including Phylum, Class, Order, Family, and Genus. The causal associations between exposures and outcomes were assessed using four established MR methods. To correct for multiple testing, the false discovery rate (FDR) method was applied. The reliability and potential biases of the results were evaluated through sensitivity analysis and F-statistics.

**Results:**

The meta-analysis incorporated a total of 20 studies that met the criteria, revealing a close association between PCOS and specific gut microbiota species. As per the results from our MR analysis, we identified six causal associations between the gut microbiome and polycystic ovary syndrome (PCOS). At the genus level, *Actinomyces* (OR_IVW_ = 1.369, *FDR* = 0.040), *Streptococcus* (OR_IVW_ = 1.548, *FDR* = 0.027), and *Ruminococcaceae UCG-005* (OR_IVW_ = 1.488, *FDR* = 0.028) were identified as risk factors for PCOS. Conversely, *Candidatus Soleaferrea* (OR_IVW_ = 0.723, *FDR* = 0.040), *Dorea* (OR_IVW_ = 0.580, *FDR* = 0.032), and *Ruminococcaceae UCG-011* (OR_IVW_ = 0.732, *FDR* = 0.030) were found to be protective factors against PCOS. Furthermore, the MR-PRESSO global test and MR-Egger regression indicated that our study results were not affected by horizontal pleiotropy (*p* > 0.05). Finally, the leave-one-out analysis corroborated the robustness of the MR findings.

**Conclusion:**

Both our meta-analysis and MR study indicates that there is a causal relationship between the gut microbiome and PCOS, which may contribute to providing novel insights for the development of new preventive and therapeutic strategies for PCOS.

## 1. Introduction

Polycystic ovary syndrome (PCOS) is the most prevalent endocrine-metabolic disorder among women of reproductive age, affecting approximately 10% of women globally, with a prevalence ranging from 8 to 18% across different populations (Lüll et al., 2020). The Andean region of Latin America exhibits the highest worldwide incidence rates (Liu et al., 2021). PCOS serves as the primary cause of anovulatory infertility in women (Glintborg and Andersen, 2010), presenting various clinical manifestations, such as chronic anovulation, hyperandrogenemia, infertility, amenorrhea, and polycystic ovarian morphology, among others. Moreover, PCOS can give rise to several metabolic abnormalities, including insulin resistance, type 2 diabetes, and obesity (Chen et al., 2014). The syndrome is linked to a multitude of comorbidities, encompassing cardiovascular diseases, mental disorders, and cancer (Luque-Ramírez and Escobar-Morreale, 2014). As a result, PCOS not only adversely affects the physical and mental well-being of women of reproductive age but also amplifies the global economic burden associated with healthcare costs. Although the etiology of PCOS remains elusive, emerging research has increasingly highlighted a strong correlation between PCOS and the gut microbiome (Thackray, 2019; Zhao et al., 2020), involving factors such as insulin resistance, metabolic syndrome, and hyperandrogenemia. Potential mechanisms by which the gut microbiota may modulate PCOS disease progression include short-chain fatty acids, the gut-brain axis, and the liver-ovary axis (Zhang et al., 2022; Liu et al., 2023). In comparison with the healthy control group, the abundance of genus Lactobacillus, Escherichia/Shigella, and Bacteroides significantly increased in the gut microbiota of PCOS patients (Guo et al., 2022). A study by Yang et al. (2021) indicated that Bacteroides could serve as a critical microbial biomarker for PCOS, even possessing diagnostic value. We have summarized past research findings, revealing that at various taxonomic levels, such as phylum, class, order, family, genus, and species, approximately hundreds of gut microbial taxa exhibit significant differences between PCOS patients and healthy individuals.

Randomized controlled trials (RCTs) are considered the gold standard for etiological research; however, their application in actual clinical research is often limited due to factors such as high costs, operational difficulties, and ethical considerations. Previous research on the relationship between gut microbiota and PCOS has primarily originated from observational studies. Researchers have examined patients’ feces or transplanted human gut microbiota into germ-free mice for experimental investigation (Quaranta et al., 2019). This process is susceptible to various factors, including species, diet, and emotions. Furthermore, due to the inherent limitations of observational studies, research findings are prone to confounding factors and reverse causality.

Mendelian randomization (MR) has emerged as a widely employed epidemiological research method in recent years (Sekula et al., 2016). As a relatively precise epidemiological research approach, MR utilizes genetic variations as instrumental variables to establish models for examining the causal relationship between exposure factors and diseases. During gamete formation, parental alleles are distributed to offspring through meiosis, a process that adheres to Mendel’s laws of segregation and independent assortment. Since genetic variations follow strict random distribution principles at conception, they are generally independent of environmental factors and precede the development of risk factors and diseases, remaining unaffected by other confounding factors. Thus, MR research methods are akin to conducting RCTs within populations. Additionally, as genetic variations are irreversible once formed, MR studies effectively avoid reverse causality (Emdin et al., 2017).

With the increasing number of large-scale genome-wide association studies (GWAS) on the gut microbiome and its relationship to various diseases (Wang et al., 2018; Kurilshikov et al., 2021), Mendelian randomization has become more widely applied in research on the gut microbiota and its association with illnesses, spanning various cancers (Long et al., 2023; Peruchet-Noray et al., 2023; Wei et al., 2023; Yuan et al., 2023), cardiovascular diseases (Meng et al., 2023), metabolic disorders (Yuan et al., 2023), immune-related diseases (Cao et al., 2023), kidney diseases (Li et al., 2023), and mental disorders (Yu et al., 2023), among others. Studies have indicated that genetic variations play a role in the development and progression of PCOS (Liu et al., 2023), ERBB4 rs192066345, YAP1 rs199505545 are considered to be closely related to PCOS (Prabhu et al., 2021). Currently, MR methods have been extensively employed in association studies between PCOS and related diseases or traits, including breast cancer, ovarian cancer, hormones, and human body composition (Chen et al., 2021; De Silva et al., 2022; Liu et al., 2022; Wang et al., 2022). However, there have been no MR reports on the causal relationship between PCOS and the gut microbiota. Although prior observational research has shown a connection between gut microbiota and the onset and progression of PCOS, the causal relationship remains unclear, This study is the first to examine the relationship between gut microbiota and PCOS through a systematic review, meta-analysis, and bidirectional two-sample Mendelian randomization study, exploring potentially influential gut microbial communities and providing new insights into the treatment and prevention of PCOS.

## 2. Materials and methods

### 2.1. Meta-analysis methods

#### 2.1.1. Search strategy

**Figure 1:**
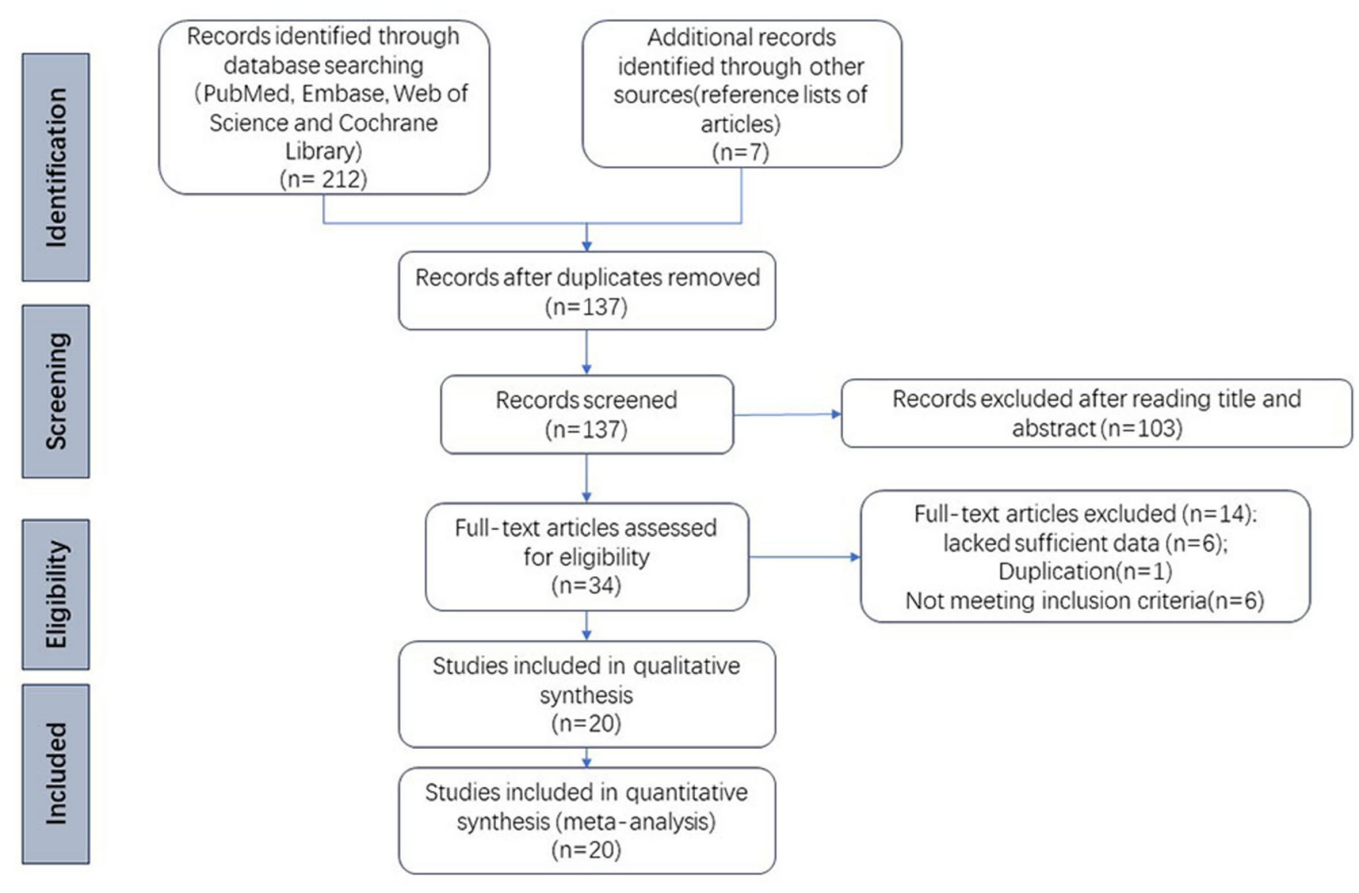
PRISMA flow diagram.

This systematic meta-analysis follows the PRISMA reporting guidelines (Page et al., 2021; Figure 1). We analyzed literature available up until June 1, 2023, sourced from four unrestricted language databases: PubMed, Cochrane Library, Web of Science, and Embase. A combination of subject words and corresponding free words were used for the search, including “Gastrointestinal Microbiomes,” “Gut Microflora,” “Gastrointestinal Microbiota,” “Polycystic Ovary Syndrome,” “Sclerocystic Ovary,” and “Ovary Syndrome, Polycystic Syndrome.” Two authors (MQS and GQ) independently carried out the literature search and selection, and in case of any disagreement regarding inclusion or exclusion, a third author (GHL) was involved in discussions to reach a resolution. Additionally, we manually reviewed and screened the reference lists of all articles to identify potential relevant studies.

#### 2.1.2. Criteria for study inclusion and exclusion

Inclusion criteria included: (Lüll et al., 2020) studies involving human subjects, including PCOS patients and healthy controls; (Liu et al., 2021) studies where fecal samples were analyzed; (Glintborg and Andersen, 2010) studies providing individual genome numbers; (Chen et al., 2014) studies where full text is available and published in English. We excluded conference papers, reviews, meta-analyses, pathological reports, and editorials, as well as animal or *in vitro* studies.

#### 2.1.3. Data extraction and quality assessment

We used a standardized data extraction list for data collection, which was recorded in an Excel spreadsheet, including study type, publication year, first author, diagnostic criteria, age range of participants, sample size and type, testing methods, and statistical results. Given that the selected articles are observational studies, we employed the “Agency for Healthcare Research and Quality (AHRQ)” for cross-sectional data quality assessment and the “Newcastle–Ottawa Scale for assessing the quality of cohort studies in meta-analysis (NOS)” for cohort studies.

#### 2.1.4. Data compilation and analysis

Given the significant discrepancies in the results of studies on gut microbial communities at different microbial levels, and the limited data available on the association between a specific type of microorganism and PCOS, a quantitative consolidated analysis could not be conducted. Therefore, we only carried out a narrative synthesis of the research results.

### 2.2. Mendelian randomization

#### 2.2.1. Study design

**Figure 2:**
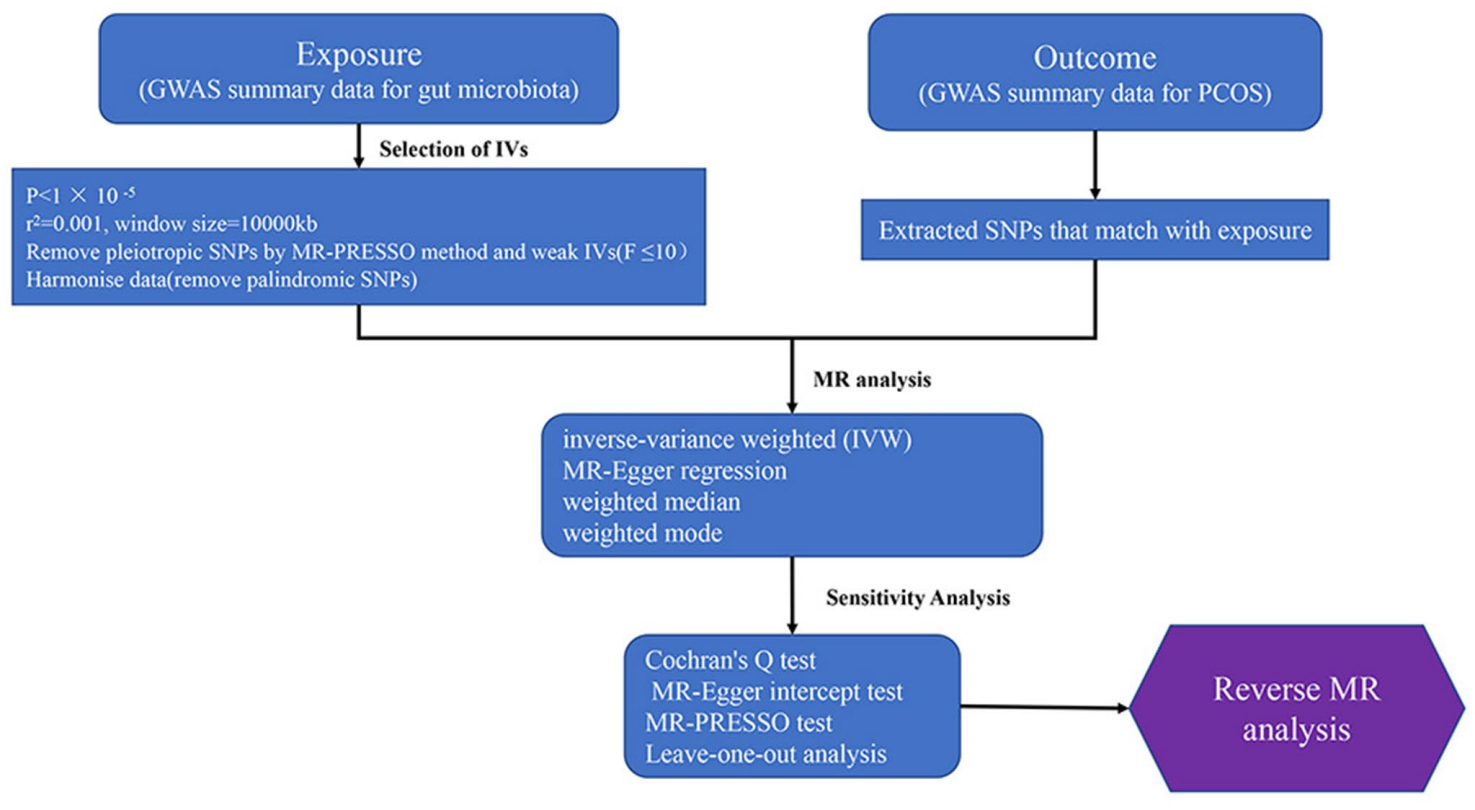
Study design and workflow (GWAS, The genome-wide association study; MR, Mendelian Randomization; PCOS, Polycystic Ovary Syndrome).

This study employs a bidirectional two-sample Mendelian randomization approach to investigate the causal relationship between the gut microbiota and PCOS, using summary data from published genome-wide association studies for analysis. In Mendelian randomization research, the instrumental variables must satisfy three core assumptions: relevance, exclusion, and independence: (1) Instrumental variables must be strongly associated with the exposure factors; (2) Instrumental variables can only affect the outcome through the exposure, and there must be no direct relationship between the instrumental variables and the outcome; (3) Instrumental variables must be independent of confounding factors that influence the relationship between exposure and outcome. The flowchart of the study is presented in Figure 2.

#### 2.2.2. Exposure and outcome data sources

The gut microbiota GWAS data is derived from the MiBioGen consortium summary statistics (Kurilshikov et al., 2021), which contains 211 taxonomic units, representing the largest and most up-to-date gut microbiome database. The database includes 9 phyla, 16 classes, 20 orders, 35 families, and 131 genera, with 15 unknown taxa excluded. The study incorporated 196 taxonomic units (9 phyla, 16 classes, 20 orders, 32 families, and 119 genera). The 16S ribosomal RNA sequencing technique was utilized in this study, encompassing a total of 24 cohorts with 18,340 participants, including 16,632 adolescents and 1,708 children. The participants were of diverse ethnicities, such as European, Middle Eastern, East Asian, American, and Canadian, with 13,266 individuals of European descent accounting for approximately 72.3% of the sample. The gut microbiome GWAS data was adjusted for age, sex, study-specific covariates, and principal components derived from population stratification.

The GWAS data for PCOS was sourced from a large-scale meta-analysis conducted by Day et al. (2019) in a European population, which included 10,074 cases and 103,164 controls. The diagnostic criteria for PCOS were based on the Rotterdam criteria, the National Institutes of Health (NIH) criteria, or self-reported diagnoses.

#### 2.2.3. Selection of genetic variants as IVs

Gut microbiota were chosen as the exposure of interest, and meaningful SNPs were selected as instrumental variables. The requirements for instrumental variables were as follows: (1) Setting a threshold of *p* < 1 × 10^−5^ to filter significant SNPs (Cao et al., 2023); (2) To ensure the independence of SNPs, the linkage disequilibrium (LD) coefficient was set at *r*^2^ = 0.001, and the LD distance were set at 10,000 kb (Davey Smith and Hemani, 2014); 3. The *F*-statistic was calculated to assess the strength of the instrumental variables (IV) in the Mendelian randomization (MR) analysis, determining the presence of weak instrument bias (Burgess et al., 2017). To further validate the association hypothesis, an *F*-statistic greater than 10 was considered to indicate a strong association (Burgess and Thompson, 2011).


$$F=\frac{N-K-1}{K}\times \frac{R^{2}}{1-R^{2}}$$

In the formula, *N* represents the sample size of the exposure factor, *R*^2^ denotes the proportion of exposure variation explained by the instrumental variable (Pierce et al., 2011), and *K* refers to the number of instrumental variables. The calculation formula for *R*^2^ is as follows:


$$R^{2}=2\times \left(1-MAF\right)\times MAF\times \beta^{2}$$

*β* represents the allele effect value, *MAF* is the minor allele frequency, and *EAF* is the effect allele frequency. *MAF* + *EAF* = 1, and MAF=min{EAF,1−EAF} . Therefore, during the calculation of *R*^2^, *EAF* can be considered equivalent to *MAF* (Davey Smith and Hemani, 2014).

#### 2.2.4. Statistical analysis

In this study, we employed a two-sample Mendelian randomization analysis to evaluate the causal association between gut microbial communities and polycystic ovary syndrome (PCOS). We used four MR analysis methods, including inverse-variance weighted (IVW), MR-Egger regression, weighted median, and weighted mode methods.

The IVW method is the primary approach for MR analysis. It first calculates the Wald ratio for each valid SNP and then combines the Wald ratios using the inverse of variance as weights, further assessing the influence of exposure factors on outcomes (Burgess et al., 2013, 2019).

The weighted median estimator provides a valid causal effect estimate even when a certain proportion (less than 50%) of invalid instrumental variables are present (Bowden et al., 2016).

MR-Egger regression fits a linear function by calculating the association of each SNP with the outcome and its association with exposure. The slope represents the estimated causal effect, and we assess the average pleiotropy based on the regression intercept (Bowden et al., 2015).

Weighted mode clusters SNPs into groups based on the similarity of individual ratio estimates, calculates the cubic variance weighting for SNPs in each cluster, and generates causal estimates based on the cluster with the highest SNP weighting (Hartwig et al., 2017; Hwang et al., 2019).

##### 2.2.4.1. Multiple testing

For the primary MR results, we applied the false discovery rate (FDR) multiple testing correction at each taxonomic level (phylum, class, order, family, and genus).

#### 2.2.5. Sensitivity analysis

Cochran’s *Q* test was employed to assess the presence of heterogeneity between the two samples (Bowden et al., 2018; Zhong et al., 2023). The MR-Egger intercept was used to determine whether there is pleiotropy among SNPs. If the intercept is greater than 0, horizontal pleiotropy is present, indicating that the outcome still exists in the absence of exposure factor interference (Brion et al., 2013; Burgess and Thompson, 2017; Yavorska and Burgess, 2017). The MR pleiotropy residual sum and outlier (MR-PRESSO) can help detect horizontal pleiotropy and identify outliers. Leave-one-out analysis involves excluding one SNP at a time, then examining the remaining SNPs using the inverse-variance weighted method to assess the influence of that SNP on the overall effect estimate. If there is no statistical difference, the MR results are considered robust.

#### 2.2.6. Reverse Mendelian randomization analysis

To investigate whether there is a causal association between PCOS and identified significant bacteria, we used PCOS as the exposure factor and the significant bacteria as the outcome. We extracted SNPs related to PCOS as IVs and conducted a reverse Mendelian randomization analysis.

All data analyses were performed using R (version 4.2.1) and the Two sample MR (version 0.5.6) package, MR–PRESSO analysis was performed by the R package “MRPRESSO” (version 1.0).

### 2.3. Ethical approval

The GWAS data utilized in this study are publicly available de-identified datasets. The Institutional Review Board (IRB) has approved these data; therefore, no additional ethical approval is required.

## 3. Results

### 3.1. Included literature and quality assessment

A total of 219 articles were retrieved from databases and other sources. After removing 82 duplicate articles and excluding 117 articles that did not meet the inclusion criteria, a total of 20 studies were included in the systematic review (Supplementary Table S3). These comprised 17 cross-sectional studies and 3 cohort studies, with a total sample size of 1,554 individuals, including 838 PCOS patients and 716 healthy controls. Four of the studies were conducted in Europe (Poland, Austria, Finland, and Spain), one in North America, 13 from Asia (12 from China, one from India), and two from Turkey, spanning both Europe and Asia. The AHQR and NOS results indicate that the literature included is of medium and high quality (Supplementary Tables S4, S5).

### 3.2. Relationship between gut microbial communities and PCOS

We summarized the changes in gut microbiota at different taxonomic levels in PCOS patients (Supplementary Tables S6–S10). There were significant differences in 154 bacterial taxa. At the phylum level, 11 taxa showed significant differences (Supplementary Table S6). Proteobacteria, Verrucomicrobia, Acidobacteria, Cyanobacteria, Gammaproteobacteria, Fusobacteria were significantly increased in PCOS patients (Zhou et al., 2020; Mammadova et al., 2021; Yu et al., 2022), while Firmicutes, Tenericutes, Bacteroidetes, and Gemmatimonadetes were significantly reduced (Lindheim et al., 2017; Jobira et al., 2020; Zhou et al., 2020; Yu et al., 2022). Jobira et al. (2020) found that Actinobacteria significantly increased in the gut of PCOS patients compared to the control group, however, the study by Yu et al. (2022) found the opposite. At the order level, 2 taxa showed significant differences (Supplementary Table S7), with an increase in Bacillales (Mammadova et al., 2021) and a decrease in ML615J-28 (Lindheim et al., 2017) in PCOS patients. At the family level, we identified 50 taxa with significant differences (Supplementary Table S8), with 37 taxa significantly enriched (Jobira et al., 2020; Mammadova et al., 2021; Zhu et al., 2021; Hassan et al., 2022; Yu et al., 2022) and 12 taxa significantly reduced (Lindheim et al., 2017; Jobira et al., 2020; Dong et al., 2021; Zhu et al., 2021; Hassan et al., 2022; Yu et al., 2022) in PCOS patients, but one bacterial taxon showed controversial results. Eyupoglu et al. (2020) found that Ruminococcaceae increased in PCOS patients, but two other studies concluded the opposite (Liu et al., 2017; Zhu et al., 2021). At the genus level, 75 taxa showed significant differences (Supplementary Table S9), with 29 taxa increasing (Liu et al., 2017; Insenser et al., 2018; Qi et al., 2019; Zhang et al., 2019; Jobira et al., 2020; Liang et al., 2020; Zhou et al., 2020; Chen et al., 2021; Mammadova et al., 2021; Hassan et al., 2022) and 44 taxa decreasing (Liu et al., 2017; Zhang et al., 2019; Zhou et al., 2020; Chen et al., 2021) in PCOS patients, while two taxa (Bacteroides and Parabacteroides) showed contradictory results in different studies (Liu et al., 2017; Qi et al., 2019; Zhang et al., 2019; Jobira et al., 2020). Finally, at the species level, 16 taxa showed significant differences (Supplementary Table S10), with 10 taxa higher in PCOS patients than in healthy controls (Torres et al., 2018; Zhang et al., 2019; Chu et al., 2020; Dong et al., 2021), 5 taxa decreased (Torres et al., 2018; Zhang et al., 2019), and one taxon (*Faecalibacterium prausnitzii*) showed inconsistent results in three studies (Torres et al., 2018; Zhang et al., 2019; Chu et al., 2020).

### 3.3. Selection of instrumental variables

At the *p* < 1 × 10^5^ level, we included 2,818 SNPs as instrumental variables (IVs), comprising 124 phylum, 223 class, 279 order, 475 family, and 1717 genus. Following linkage disequilibrium (LD) clumping and harmonization, the number of candidate IVs associated with specific bacterial taxa for each outcome ranged from 4 to 22. It was found that all instrumental variables had an *F*-statistic >10. Using the MR-PRESSO global test (*p* > 0.05) and MR-Egger regression (*p* > 0.05), the results indicated no horizontal pleiotropy for the SNPs used as IVs, suggesting that there was no confounding in this study. Detailed IV information is listed in Supplementary Table S1.

### 3.4. Causal influence of gut microbiota on PCOS

**Figure 3:**
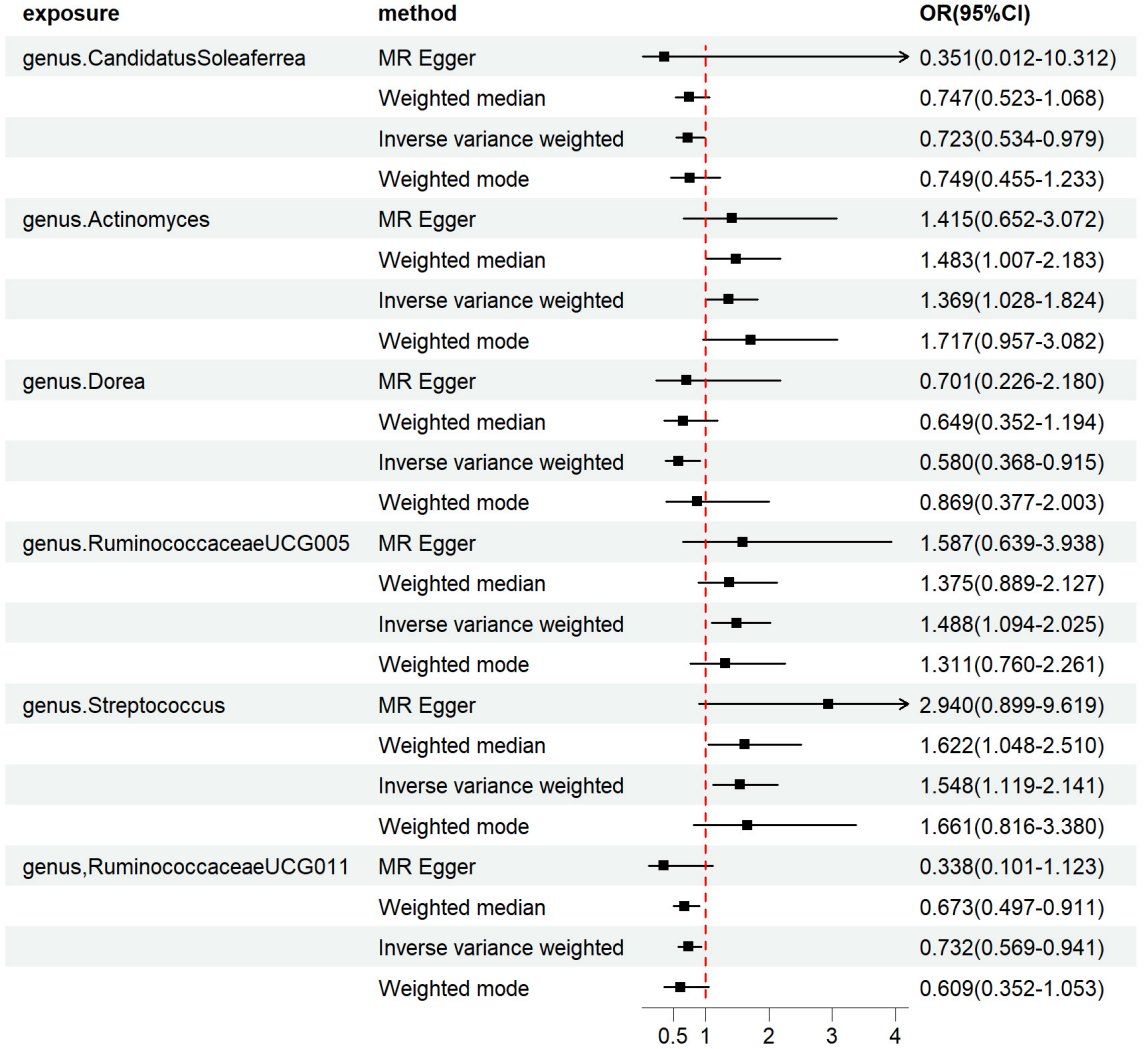
Forest plots of the MR results of the four identified causal associations: The figure showed the IVW estimates of significantly PCOS-associated gut microbiota taxa. The black square represent the IVW estimates, and the black bars represent the 95% confidence intervals of IVW estimates. The OR > 1 indicates increased risk while<1 indicates decreased risk.

**Figure 4:**
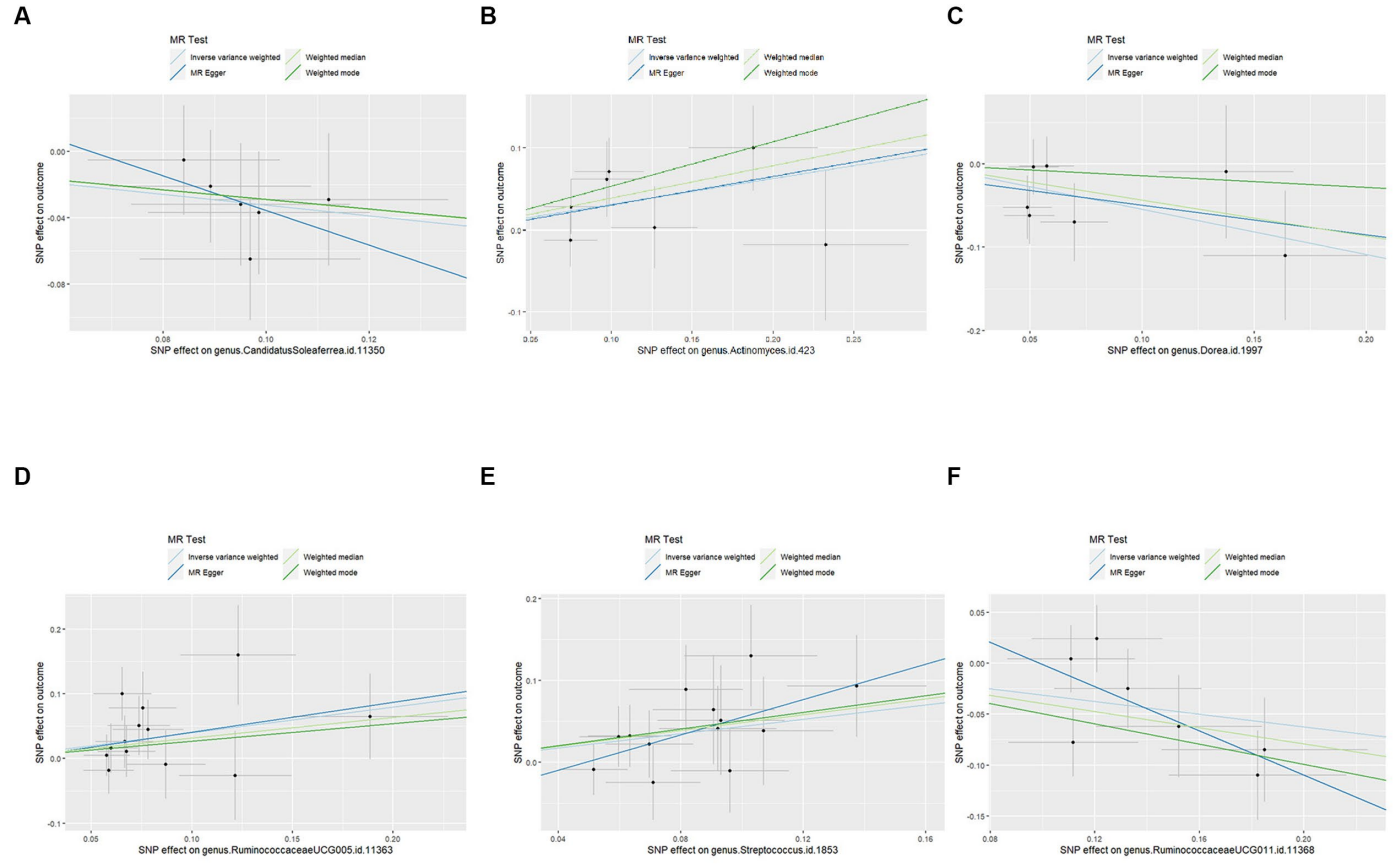
Scatter plots for the causal effects of the identified bacterial taxa on PCOS: **(A)** the causal effect of the genus CandidatusSoleaferrea on PCOS; **(B)** the causal effect of the genus Actinomyces on PCOS; **(C)** the causal effect of the genus Dorea on PCOS; **(D)** the causal effect of the genus RuminococcaceaeUCG005 on PCOS; **(E)** the causal effect of the genus Streptococcus on PCOS; **(F)** the causal effect of the genus RuminococcaceaeUCG011 on PCOS; Lines that diagonally ascend from left to right suggest a positive correlation, implying a facilitating role of gut microbiota in the progression of PCOS. The corresponding horizontal and vertical lines delineate the 95% confidence interval for each correlation. Conversely, lines that descend diagonally from left to right signal a negative correlation, indicating that the gut microbiota exerts an inhibitory effect on PCOS (MR, Mendelian randomization; SNPs, single nucleotide polymorphisms; PCOS, Polycystic Ovary Syndrome).

We employed four classical MR methods for analysis. At the phylum, class, and order levels, we did not find a causal association between gut microbiota and PCOS. However, at the genus level, we identified six causal associations between gut microbial communities and PCOS. We estimated the odds ratio using the Inverse Variance Weighting (IVW) method, with denoting this as the “OR_IVW_.” *Actinomyces* (OR_IVW_ = 1.369, 95%CI:1.028–1.824, *FDR* = 0.040), *Streptococcus* (OR_IVW_ = 1.548, 95%CI:1.119–2.141, FDR = 0.027), and *RuminococcaceaeUCG005* (OR_IVW_ = 1.488, 95%CI: 1.094–2.025, *FDR* = 0.028) were identified as risk factors for PCOS. Meanwhile, *Candidatus Soleaferrea* (OR_IVW_ = 0.723, 95%CI: 0.534–0.979, *FDR* = 0.040), *Dorea* (OR_IVW_ = 0.580, 95%CI: 0.368–0.915, *FDR* = 0.032), and *RuminococcaceaeUCG011* (OR_IVW_ = 0.732, 95%CI: 0.569–0.941, *FDR* = 0.030) were identified as protective factors for PCOS (Table 1). Furthermore, MR-Egger, weighted median estimator, and weighted mode estimator methods produced causal effect estimates with similar magnitudes and directions as the aforementioned IVW method (Figures 3, 4).

**Table 1:** Four MR models’ estimation of the causal relationships between identified bacterial taxa and PCOS and tests for heterogeneity and horizontal pleiotropy.

Exposure (genus)	nSNP	Method	OR (95%CI)	*p*-value	*P_heterogeneity_*	*P_pleiotropy_*	*P_MR-PRESSO_*
CandidatusSoleaferrea	6	MR Egger	0.351 (0.012–10.312)	0.580	0.878	0.696	0.939
		Weighted median	0.747 (0.523–1.068)	0.110			
		IVW	0.723 (0.534–0.979)	*FDR* (0.040)	0.927		
		Weighted mode	0.749 (0.455–1.233)	0.310			
genus. Actinomyces	7	MR Egger	1.415 (0.652–3.072)	0.420	0.441	0.931	0.594
		Weighted median	1.483 (1.007–2.183)	0.050			
		IVW	1.369 (1.028–1.824)	*FDR* (0.040)	0.568		
		Weighted mode	1.717 (0.957–3.082)	0.120			
Dorea	7	MR Egger	0.701 (0.226–2.180)	0.570	0.574	0.735	0.724
		Weighted median	0.649 (0.352–1.194)	0.170			
		IVW	0.580 (0.368–0.915)	*FDR* (0.032)	0.683		
		Weighted mode	0.869 (0.377–2.003)	0.750			
RuminococcaceaeUCG005	13	MR Egger	1.587 (0.639–3.938)	0.340	0.525	0.886	0.649
		Weighted median	1.375 (0.889–2.127)	0.150			
		IVW	1.488 (1.094–2.025)	*FDR* (0.028)	0.608		
		Weighted mode	1.311 (0.760–2.261)	0.350			
Streptococcus	13	MR Egger	2.940 (0.899–9.619)	0.100	0.889	0.294	0.871
		Weighted median	1.622 (1.048–2.510)	0.030			
		IVW	1.548 (1.119–2.141)	*FDR* (0.027)	0.859		
		Weighted mode	1.661 (0.816–3.380)	0.187			
RuminococcaceaeUCG011	7	MR Egger	0.338 (0.101–1.123)	0.137	0.264	0.254	0.248
		Weighted median	0.673 (0.497–0.911)	0.010			
		IVW	0.732 (0.569–0.941)	*FDR* (0.030)	0.197		
		Weighted mode	0.609 (0.352–1.053)	0.126			

### 3.5. Sensitivity analysis

**Figure 5:**
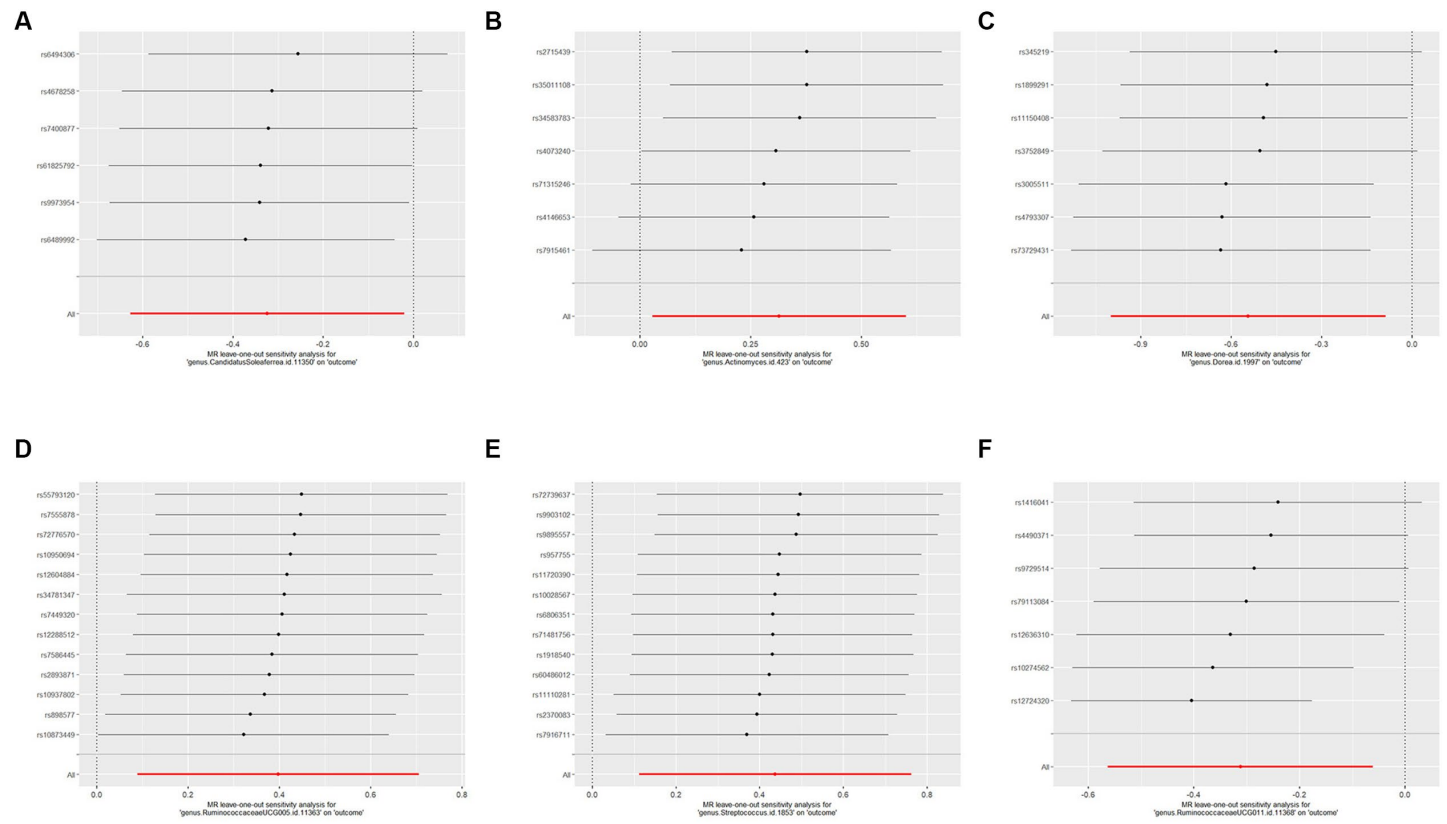
Forest plots of leave-one-out sensitivity analysis for the six identified associations: **(A)** MR leave-one-out sensitivity analysis for CandidatusSoleaferrea on PCOS; **(B)** MR leave-one-out sensitivity analysis for Actinomyces a on PCOS; **(C)** MR leave-one-out sensitivity analysis for Dorea on PCOS; **(D)** MR leave-one-out sensitivity analysis for RuminococcaceaeUCG005 on PCOS; **(E)** MR leave-one-out sensitivity analysis for Streptococcus on PCOS; **(F)** MR leave-one-out sensitivity analysis for RuminococcaceaeUCG011 on PCOS. These plot represents the leave-one-out sensitivity analysis performed to evaluate the influence of each individual SNP on the pooled effect size. Each point on the plot corresponds to the recomputed pooled effect size when the respective SNP on the Y-axis is omitted from the meta-analysis. The horizontal lines represent the 95% confidence intervals of the recalculated effect sizes. If the omission of any individual study leads to a significant shift in the pooled effect size, it suggests that the particular study has an undue influence on the overall meta-analysis results.

We used IVW and MR-Egger methods for heterogeneity testing, and the results found no heterogeneity between the IVs (*p* > 0.05; Table 1). The MR-Egger regression intercepts were not significantly deviated from 0, indicating no horizontal pleiotropy (*p* > 0.05). The MR-PRESSO global test confirmed the same results (*p* > 0.05), suggesting that there was no confounding in this study. Leave-one-out analysis indicated that the MR findings were robust (Figure 5).

### 3.6. Bidirectional MR findings

To assess the reverse causal effect, we performed bidirectional Mendelian randomization analysis with PCOS as the exposure and the six identified significant gut microbial taxa as the outcomes. None of the four MR methods detected significant causal estimates, indicating no causal association between PCOS and the identified bacterial taxa. The results of the reverse MR analysis are shown in Supplementary Table S2.

## 4. Discussion

To the best of our knowledge, this is the first study to investigate the causal association between gut microbiota and PCOS using bidirectional Mendelian randomization. Simultaneously, this study compiles the latest literature for meta-analysis. We utilized the largest and most recent gut microbiome GWAS data and PCOS GWAS data, with closely related SNPs as instrumental variables. Through bidirectional Mendelian randomization analysis, we identified six causal associations between gut microbial taxa and PCOS. Our sensitivity analysis showed no presence of horizontal pleiotropy, indicating that our MR analysis was not affected by confounding factors. The leave-one-out test confirmed the robustness of our bidirectional MR study.

The meta-analysis indicates that there are specific microbial communities at the phylum, order, family, genus, and species levels that are closely associated with PCOS, involving a total of 154 bacterial taxa. The results of our Mendelian Randomization (MR) analysis confirm the existence of a causal relationship between gut microbiota and PCOS disease. A previous study on PCOS employed 16S rRNA sequencing of the V3-V4 region and found a higher abundance of *Streptococcus* in the intestines of PCOS patients than in healthy individuals. In addition, this study found that androgen levels and BMI were significantly increased in PCOS patients with elevated *Streptococcus* abundance (Liu et al., 2017). These findings suggest that *Streptococcus* may be involved in the development of PCOS, and our study provides similar results. In our study, *Streptococcus* was identified as a risk factor for PCOS (OR_IVW_ = 1.548, *FDR* = 0.027), with statistically significant differences. It has been reported that the abundance of *Dorea* in the intestines of PCOS patients is significantly reduced compared to healthy individuals (*p* = 0.005)(Chen et al., 2021). Yin et al. (2022) found that *Dorea* was significantly higher in the intestines of healthy controls compared to PCOS patients among non-obese individuals, suggesting that *Dorea* may play a protective role in the pathogenesis and development of PCOS. Our MR results also indicated *Dorea* as a protective factor for PCOS (OR_IVW_ = 0.580, *FDR* = 0.032), consistent with this finding. However, another study found that *Dorea* was significantly more abundant in the intestines of prediabetic PCOS patients than in healthy individuals (*FDR* = 0.03), suggesting that *Dorea* may increase the risk of developing metabolic subtypes of PCOS. *Candidatus Soleaferrea* is known to exert gut-protective effects through the secretion of metabolites (Cai et al., 2020). A study comparing the gut microbiota of 12 obese PCOS patients (BMI ≥ 24 kg/m^2^) and 19 non-obese PCOS patients (BMI < 24 kg/m^2^) found that *Candidatus Soleaferrea* was significantly more abundant in non-obese PCOS patients, suggesting that it may reduce the risk of obesity in PCOS patients (Li et al., 2022). However, no studies have reported on the causal relationship between *Candidatus Soleaferrea* and PCOS to date. In our study results, *Candidatus Soleaferrea* significantly reduced the risk of PCOS (OR_IVW_ = 0.723, *FDR* = 0.040).

We also found that *Ruminococcaceae UCG011* reduced the risk of PCOS, while *Actinomyces* and *Ruminococcaceae UCG005* increased the risk. However, there is currently no direct evidence linking *Actinomyces*, *Ruminococcaceae UCG005*, or *Ruminococcaceae UCG011* specifically with PCOS.

It has been reported that *Ruminococcaceae UCG011* is positively correlated with acetate concentration (Zhang et al., 2022). Acetate, a short-chain fatty acid (SCFA) derived from gut microbiota, has various important functions in the human body, such as providing energy, maintaining gut health, and regulating immune responses. We hypothesize that *Ruminococcaceae UCG011* may reduce the risk of PCOS by increasing acetate concentration. *Actinomyces* is a controversial gut microbe, with some studies suggesting it is beneficial to human health (Cani et al., 2007), while others hold the opposite view. Animal studies have found that high-fat diets lead to a significant increase in intestinal *Actinomyces* abundance (Li et al., 2020). *Ruminococcaceae UCG005* is generally considered a protective gut bacterium due to its ability to produce short-chain fatty acids (butyrate; Chen et al., 2021) and induce beneficial metabolic effects by enhancing mitochondrial activity, improving energy metabolism, and activating intestinal gluconeogenesis (Hartstra et al., 2015). A study of 2,166 participants found a negative correlation between *Ruminococcaceae UCG005* abundance and the risk of insulin resistance and type 2 diabetes (*β* = −0.09; 95% CI: −0.13 to −0.05; *p* < 0.001). However, our study suggests that *Ruminococcaceae UCG005* is a risk factor for PCOS (OR_IVW_ = 1.488, *FDR* = 0.028). Since there is currently no research on the relationship between *Actinomyces*, *Ruminococcaceae UCG005,* and PCOS, our study provides a reference for further exploration.

Gut microbiota can promote the induction or development of PCOS through various mechanisms, but the exact mechanism by which gut microbial communities cause PCOS has not yet been determined. Many dietary components can influence disease onset and progression by targeting gut microbiota (Tao et al., 2020). Vitamin D is known to promote calcium homeostasis and bone growth, and aids in preventing or mitigating inflammation and immune-mediated tissue damage (Murdaca et al., 2019). Chronic inflammation is one of the significant pathogenic mechanisms of Polycystic Ovary Syndrome (PCOS), and patients with PCOS are often accompanied by Vitamin D deficiency (Mu et al., 2021). It has been reported that a deficiency in Vitamin D intake can lead to an increase in the quantity of Bacteriodetes (Yamamoto and Jørgensen, 2020). Furthermore, the abundance of Bacteriodetes in the gut of PCOS patients is higher compared to healthy individuals. Therefore, we boldly hypothesize that Vitamin D might influence the occurrence of PCOS by affecting the composition of the gut microbiota. It has been found that people on high-fat diets have reduced bacterial diversity in their gut (Schnorr et al., 2014). In many developing countries, obesity caused by high-fat diets is gradually increasing (Goss et al., 2014; Yang and Yu, 2018). Considering the complex relationship between diet, gut microbiota, and PCOS, more research and mediation MR are needed in the future to further uncover their associations and mechanisms (Carter et al., 2021).

There are some limitations to this study. First, GWAS are unlikely to explain all genetic traits of complex phenotypes (Altshuler et al., 2008). Human behavior is complex, and although understanding the genetic risk of disease can help prevent its occurrence to some extent, environmental factors themselves also play a role in the development of disease (Meisel et al., 2015). Furthermore, environmental factors can influence disease by affecting genetics (Agustí et al., 2022), and MR can only eliminate the interference of confounding factors, such as the environment, to a certain extent. Second, the GWAS data for PCOS in our study comes from European ancestry, and although most of the gut microbiota GWAS data comes from European ancestry, a small portion is from other ethnic populations, which may cause some bias in the results. Additionally, there is an inconsistency between the populations studied in the meta-analysis and the MR analysis. The meta-analysis incorporated studies from multiple ethnicities, predominantly Asian, while the MR analysis was conducted on a European population. Finally, due to the large number and significant differences in the types of microbial communities involved in the included studies, and the small number of studies focusing on a particular community, our meta-analysis cannot perform quantitative analysis.

## 5. Conclusion

By providing a comprehensive assessment of the causal relationship between gut microbiota and PCOS, our study contributes valuable insights into the potential role of gut microbes in the development and progression of this condition. Future research should aim to further elucidate the underlying mechanisms by which these microbial communities influence PCOS and explore potential therapeutic strategies targeting the gut microbiota to mitigate the disease’s impact on affected individuals.

## Data availability statement

This study analyzed publicly available datasets. The primary summary statistics for gut microbiome can be downloaded from: https://mibiogen.gcc.rug.nl/, and the PCOS dataset is accessible at: https://www.repository.cam.ac.uk/handle/1810/289950.

## Ethics statement

The study utilized publicly available de-identified data from participant research approved by the ethics standard committees. Therefore, no additional separate ethical approval was required for this research.

## Author contributions

QM conceived the study, conducted data analysis, and authored the manuscript. HG contributed to the experimental design and participated in the revision of the manuscript. QG provided valuable assistance in data analysis. MX was involved in the design of experimental methods and provided critical review and editing of the manuscript. All authors contributed to the article and approved the submitted version.

## Funding

This research was supported by the National Natural Science Foundation of China (82104913) and the National Administration of Traditional Chinese Medicine National Renowned TCM Master Studio Construction Project (1199ws02).

## Conflict of interest

The authors declare that the research was conducted in the absence of any commercial or financial relationships that could be construed as a potential conflict of interest.

## Publisher’s note

All claims expressed in this article are solely those of the authors and do not necessarily represent those of their affiliated organizations, or those of the publisher, the editors and the reviewers. Any product that may be evaluated in this article, or claim that may be made by its manufacturer, is not guaranteed or endorsed by the publisher.
